# Role of Versican, Hyaluronan and CD44 in Ovarian Cancer Metastasis

**DOI:** 10.3390/ijms12021009

**Published:** 2011-01-31

**Authors:** Miranda P. Ween, Martin K. Oehler, Carmela Ricciardelli

**Affiliations:** 1 Research Centre for Reproductive Health, School of Paediatrics and Reproductive Health, Robinson Institute, University of Adelaide, Adelaide, South Australia 5005, Australia; E-Mails: miranda.ween@adelaide.edu.au (M.P.W.); martin.oehler@adelaide.edu.au (M.K.O.); 2 Research Centre for Infectious Diseases, School of Molecular Biosciences, University of Adelaide, South Australia 5005, Australia; 3 Department of Gynaecological Oncology, Royal Adelaide Hospital, Adelaide, South Australia 5000, Australia

**Keywords:** extracellular matrix, hyaluronan, versican, CD44, adhesion, metastasis

## Abstract

There is increasing evidence to suggest that extracellular matrix (ECM) components play an active role in tumor progression and are an important determinant for the growth and progression of solid tumors. Tumor cells interfere with the normal programming of ECM biosynthesis and can extensively modify the structure and composition of the matrix. In ovarian cancer alterations in the extracellular environment are critical for tumor initiation and progression and intra-peritoneal dissemination. ECM molecules including versican and hyaluronan (HA) which interacts with the HA receptor, CD44, have been shown to play critical roles in ovarian cancer metastasis. This review focuses on versican, HA, and CD44 and their potential as therapeutic targets for ovarian cancer.

## 1. Introduction

Ovarian cancer is the most lethal gynecological cancer and ranks as the fifth most common cause of cancer-related death in women in the Western world [1,2]. Over 70% of women present with advanced disease and despite aggressive treatment, the 5 year survival rate of patients with ovarian carcinoma is only 30–50%. This poor prognosis results from late diagnosis and ineffective therapy for advanced stage disease. A major challenge for ovarian cancer research remains the identification of tumor markers to aid in early diagnosis and the identification of novel therapeutic strategies for advanced disease. Significant improvements in ovarian cancer survival will require the development of more effective molecularly targeted diagnostics and/or therapeutics.

## 2. Ovarian Cancer Metastasis

Ovarian carcinomas are believed to spread by detaching from the surface of the ovary and attaching to and invading the peritoneum which lines the organs of the abdominal cavity. Involvement of the peritoneum predicates an adverse situation for the patient which impacts significantly on prognosis. This is evidenced by the fact that stage I patients have 5 and 10 year survival rates of over 90%, whereas patients with stages III disease have a 5 year survival of only about 30% [3,4]. Ovarian cancer cells are anatomically placed to metastasize into the peritoneal cavity. However it is not clear whether the anatomical location is solely responsible for intra-peritoneal spread of ovarian carcinomas or whether other factors may foster the implantation of cancer cells onto the peritoneal lining. Peritoneal cells are known to express and secrete several extracellular matrix (ECM) proteins, adhesion molecules and many other factors that provide an important microenvironment to aid the implantation of ovarian cancer cells [5–11]. Alterations in the extracellular microenvironment surrounding ovarian cancer cells are therefore believed to be critical for tumor initiation, progression and peritoneal tumor spread. Indeed, it has been demonstrated that peritoneal cells produce both extracellular matrix molecule, hyaluronan (HA) and the HA receptor, CD44 [12–14], and can promote ovarian cancer motility by increasing their HA production in co-culture with ovarian cancer cells [14].

## 3. Role of the Extracellular Matrix in Ovarian Cancer Metastasis

The extracellular matrix (ECM) is a highly organized three-dimensional structure with many physiological and pathological roles. The ECM, in addition to maintaining tissue integrity, also regulates cell migration, cellular differentiation, and proliferation and also provides a reservoir of cytokines and growth factors. Tumor cells interfere with the normal programming of ECM biosynthesis and can extensively modify the structure and composition of the matrix [15]. There is increasing evidence to suggest that ECM components play an active role in tumor progression and are important determinants for the growth and progression of solid tumors [16]. The extensive remodeling of the normal ECM in tumors can proceed through the degradation of pre-existing ECM molecules and/or by the synthesis of ECM components, which in many cases are not present in the ECM of normal tissues. Altered expression of several ECM molecules including versican, hyaluronan (HA) and CD44 have been described in ovarian cancer and impact on ovarian cancer outcome [17]. Table 1 summarizes the studies which have investigated the expression of HA, the HA degrading enzymes hyaluronidases, CD44 and versican in ovarian cancer progression and their functional role on ovarian cancer cells.

### 3.1. Role of HA in Ovarian Cancer

HA is a large polymer which extrudes into the extracellular space and is made up of repeating disaccharides of N-acetyl glucosamine and d-glucuronic acid with a varying molecular weight and size depending on the tissue in which it is found [51]. HA is synthesized at the surface of cells by one of three HA synthases (HAS 1, 2, or 3) and plays a role in various cellular functions including adhesion, motility and differentiation [52]. The physiological function of HA varies greatly depending on HA size, and the presence or absence of HA binding proteins and cell surface receptors [53,54]

**Figure 1.**
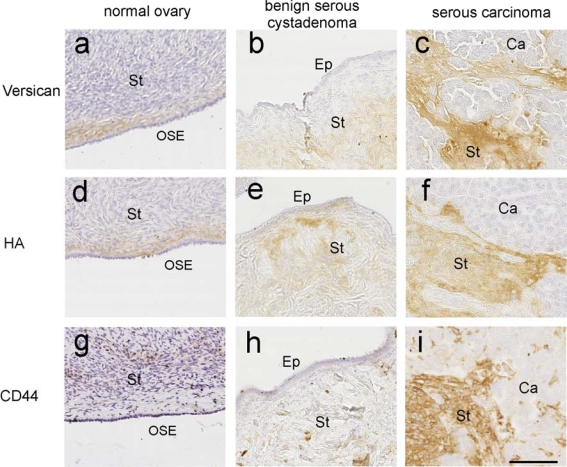
Co-localization of versican, HA and CD44 in ovarian tissues. Normal ovary (a, d, g), benign serous cystadenoma (b, e, h) and serous ovarian carcinoma (c, f, i). Formalin fixed paraffin sections (5 μm) were immunostained with versican (Vc, 1/500, a, b, c) provided by Assoc Prof Richard Le Baron (Division of Life Science, University of Texas at San Antonio, San Antonio, TX) previously described [76] following digestion with chondroitinase ABC. HA was detected using biotinylated HABP (d, e, f) as described previously [77]. CD44 immunohistochemistry (g, h, i) was achieved using mouse anti-CD44 (1/800 Clone 156-3C11, Neomarkers, Fremont, USA), with citrate buffer microwave retrieval. bar = 100 μm. All images are at the same magnification. Strong stromal (St) staining for versican, HA and CD44 is present in serous ovarian carcinoma tissue. In comparison, lower versican, HA and CD44 stromal staining is present in the stroma of normal ovary and benign serous cystadenoma tumor tissues. No versican or HA staining was detected in the ovarian surface epithelium (OSE), benign epithelial cells (Ep) or serous ovarian carcinoma cells (Ca).

Many tumors are enriched in HA [53,55]. HA levels can be increased within the tumor cells themselves or within the tumoral stroma [20,56]. Increased HA has been shown to be closely correlated with the degree of invasiveness and metastatic potential in ovarian cancer tumor models [19,22]. Accumulation of high levels of HA have been found to be associated with reduced levels of the hyaluronidase HYAL1 [28]. High levels of stromal HA are associated with a poorer prognosis in patients with breast cancer, non-small-cell lung cancer, colon cancer, prostate cancer and ovarian cancer [20,55–60]. In ovarian cancer patients, high HAS1 levels in ovarian cancer cells but not HAS2 or HAS3 are associated with reduced overall survival [24]. This is consistent with other reports describing high levels of stromal HA and HAS1 to be independent predictors of overall survival [20,24]. Our recent studies have confirmed the presence of high HA levels in the peritumoral stroma of serous ovarian carcinomas which correlate with CD44 and versican expression (Figure 1).

### 3.2. CD44—A Key Receptor for HA

The CD44 glycoprotein is an acidic molecule whose charge is largely determined by sialic acid. CD44 is a multi-structural cell surface receptor which binds HA with a particularly high affinity [61,62]. It belongs to a family of transmembrane glycoproteins which contain a variable extracellular domain, a 23-amino acid transmembrane domain, and a 70-amino acid cytoplasmic domain [63]. Up to ten different CD44 isoforms have been documented due to differential splicing of the 10 variant exons [64,65]. The most common isoform is the standard CD44 (CD44s), in which exon 5 is spliced directly to exon 16 resulting in an approximately 85 kDa glycoprotein.

### 3.3. The Role of CD44 and Its Interactions with HA in Cancer

The binding of HA to CD44 triggers direct cross-signaling between different signaling pathways including HER2, c-src kinase and ERK [21,33,37,43,44,66] and it is this function which is thought to be involved in increased motility, adhesion, and invasion of cancer cells as well as tumor growth, including ovarian cancer [13,32,61,67,68]. High CD44 levels have been associated with unfavorable prognosis in a variety of cancers including those of the breast [69], stomach [70], head and neck [71], biliary tract [72], and prostate [73]. Ovarian cancers express standard and variant isoforms of CD44 [74]. Higher CD44s expression was shown in ovarian cancer compared with benign and borderline tumors [75].

Several studies have suggested that patients with CD44 positive tumors have a significantly shorter disease-free survival than patients with CD44 negative tumors [31,34,38]. In contrast, other studies have demonstrated that high CD44s expression is associated with improved ovarian cancer outcome [39–41], whilst other studies have found no association between CD44s or CD44 variant expression with ovarian cancer metastasis or survival outcome [74,78,79].

Differences between the studies could be attributed to technical factors like the use of different antibodies and different detection methods. Furthermore, the cohorts of ovarian cancer patients examined in the various studies were highly heterogeneous and composed of patients with variable tumor types, stages and treatment regimes. Our recent studies have confirmed the presence of high CD44 immunostaining particularly in the peritumoral stroma of serous ovarian carcinomas which was associated with high HA and versican expression (Figure 1).

### 3.4. Versican—An Interacting Partner of HA

Versican is a large HA binding proteoglycan detected in increased quantities in tumor lesions [80]. It is a member of the large aggregating chondroitin sulfate (CS) hyalectins family which includes aggrecan, neurocan, and brevican [81]. Versican consists of an N-terminal HA-binding G1 domain, a chondroitin sulfate binding region, and a C-terminal, G3 domain [82]. The G1 and G3 domains bind specific proteins and have differing domains and motifs which bind to a wide range of molecules including fibronectin, and tenascin [83]. Alternative splicing produces five known isoforms of versican [84,85].

### 3.5. Versican Is Associated with Poor Outcome

Elevated levels of versican have been reported in most malignancies, including brain tumors, melanomas, osteosarcomas, lymphomas and cancer of the breast, prostate, colon, lung, pancreas, endometrium, and ovary [46–48,75,86–98]. Versican expression is also associated with cancer relapse and poor patient outcome in breast, prostate and many other cancer types including ovarian cancer [80]. Cultured mammary and prostate fibroblasts produced significant amounts of versican [86,87]. Furthermore, it was demonstrated that breast and prostate cancer cells could increase versican production by stromal cells [86,87].

In ovarian cancer, versican has been shown to be present in both cancer cells and the peritumoral stroma and increased expression relates with tumor progression and poorer survival outcome [46,49,88]. High expression of versican in peritumoral stroma was associated with advanced stage, large residual disease, serous histological type, and reduced 5-year survival [46]. Increased expression of versican was also identified as a key protein involved in ovarian cancer metastasis [48]. Elevated levels of versican have been observed in primary ovarian tumors and secondary metastases when compared with normal ovaries [47]. Immunohistochemistry studies in our laboratory have confirmed that versican is increased in the stromal compartment of serous ovarian carcinoma compared to the stroma of normal ovaries or benign ovarian tumors (Figure 1).

### 3.6. Role of Versican and HA in Promoting Cancer Cell Motility and Invasion

**Figure 2.**
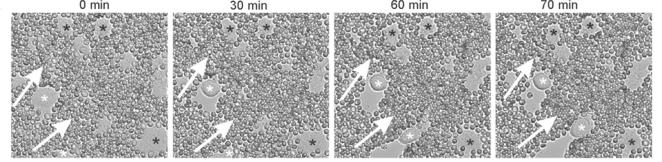
Pericellular matrix formation in motile ovarian cancer SKOV-3 cells. The confluent SKOV-3 monolayer was wounded and treated with versican containing media (0.5 U/mL) for 4 h. The white asterisks indicate motile SKOV-3 cells with a polar pericellular matrix observed over a 70 min time period, using a red blood cell exclusion assay. The black asterisks indicate non-motile SKOV-3 cells lacking a pericellular matrix. The white arrows indicate the direction of cell movement. Red blood cells diameter = 7 m. From Ween *et al.* [26].

HA becomes deposited in the tissue spaces immediately surrounding invasive tumors and together with other ECM components, forms a protective pericellular coat around the cells [89]. The assembly of pericellular matrix rich in HA and versican is a prerequisite for proliferation and migration of mesenchymal cells [90] and has recently been shown to promote the motility of prostate cancer cells [91]. Our most recent data also shows that motile SKOV-3 ovarian cancer cells expressing CD44 also form a HA/versican pericellular matrix [26] (Figure 2). Furthermore, we demonstrated that versican treatment can induce ovarian cancer cell invasion through an ECM barrier [26]. These findings are also supported by a recent study demonstrating that versican-treated ovarian cancer cells have increased invasive potential [88]. Our results suggest that formation of a CD44/HA/versican macromolecular complex promotes the motility and invasion of ovarian cancer cells. The dependence of CD44 for the versican and HA-mediated effects was confirmed by the inhibition of pericellular matrix formation as well as versican mediated motility and invasion of ovarian cancer cells following treatment with CD44 neutralizing antibody [26].

### 3.7. Involvement of HA and CD44 in the Adhesion of Ovarian Cancer Cells to Peritoneal Cells

Ovarian cancer cell adhesion to mesothelial cell monolayers is mediated at least in part by the interaction between HA and CD44 [6,92]. It has also been suggested that ovarian cancer cell interactions with mesothelial cell HA may also mediate tumour metastasis [93]. The addition of anti-CD44 antibodies has been reported to significantly decrease adhesion of ovarian cancer cells to HA [6]. *In vivo* studies have suggested that CD44s is required for human ovarian cancer cell adhesion to mesothelial cell surface HA [12,29]. Our own *in vitro* studies have shown that HA increases the adhesion of CD44 expressing ovarian cancer cells to peritoneal cells [26].

**Figure 3.**
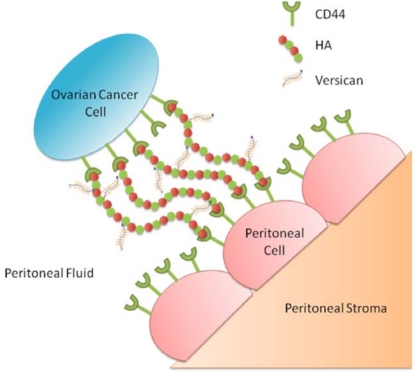
Proposed model of HA, CD44 and versican interactions between ovarian cancer and peritoneal cells. The formation of a stabilized HA/versican pericellular matrix surrounding ovarian cancer cells increases motility and protects the ovarian cancer cells against the mechanical forces in the peritoneal cavity and enable ovarian cancer cells to strongly adhere to CD44 expressed on peritoneal cells. This allows subsequent ovarian cancer invasion and peritoneal dissemination. From Ween *et al.* [26].

Overall the combined data supports a role for versican together with HA and CD44 in a number of the key steps needed for ovarian cancer metastasis. Our working model proposes that versican from the peritumoral stroma binds HA in the ECM (Figure 3). The formation of a stabilized HA/versican pericellular matrix surrounding ovarian cancer cells, protects the ovarian cancer cells against the mechanical forces in the peritoneal cavity and enables strong ovarian cancer cell adhesion to CD44 expressed by peritoneal cells. This provides the basis for subsequent ovarian cancer dissemination throughout the abdominal cavity.

## 4. Cancer Therapies Targeting Extracellular Components

### 4.1. HA Therapies

Because of the ability to bind cell surface CD44, which is upregulated in many cancer types, HA has been conjugated or co-admininistered with traditional chemotherapy drugs to increase the direct targeting of the drug to the cancer cells. More pronounced cytotoxic effects were observed when HA was conjugated to paclitaxel on CD44-overexpressing breast cancer cells compared to CD44-deficient cells, suggesting that HA-conjugation can be potentially utilized as tumor-targeted therapy [94]. Conjugated HA-cisplatin has also been shown to be effective in targeting breast cancer cells in an orthotopic mouse model [95].

In ovarian cancer cells, paclitaxel-HA conjugate interacted with CD44, entered the cells through a receptor-mediated mechanism, and exerted a concentration-dependent inhibitory effect on tumor cell growth [96]. Furthermore, after intra-peritoneal administration in mice HA bioconjugate distributed uniformly within the peritoneal cavity, was well tolerated, and not associated with local toxicity [96]. Blood levels of paclitaxel in mice treated with HA-conjugated paclitaxel were also much higher and persisted longer than those obtained with the unconjugated paclitaxel [96]. Paclitaxel-HA resulted in a 2.5-fold increase in therapeutic activity when compared with paclitaxel alone [96] and decreased tumor burden in ovarian cancer xenograft mice [97].

HA has also been investigated as a potential conjugate with other types of cancer therapies besides chemotherapy drugs. When polyethylenimine (PEI) DNA particles are conjugated with HA they showed more specific targeting of breast cancer cells with high CD44 expression [98]. A siRNA/PEI-HA complex exhibited higher CD44 gene silencing efficiency in melanoma cells compared to siRNA/PEI complex alone [99] Similarly, liposomes-protamine-HA nanoparticles used for systemic delivery of siRNA into melanoma cells had a broader effective therapeutic dose range than nanoparticles without HA [100].

Methods of eliminating HA in tumors using hyaluronidase enzymes to degrade the HA have also been investigated. Pilot clinical studies with a bovine hyaluronidase preparation examined whether this enzyme could increase anticancer drug action in combination therapies [101]. However, despite early signs of clinical activity, systemic administration of the bovine hyaluronidase elicited allergic reactions which, combined with a poor plasma half-life, limited clinical efficacy to local-regional chemotherapy in bladder carcinoma [102]. A pegylated variant of human hyaluronidase PH20, PEGPH20 with an increased half life has recently been evaluated in prostate cancer xenograft models [103]. In PC3 prostate tumors, i.v. administration of PEGPH20 depleted tumor HA and significantly inhibited tumor growth. PEGPH20 treatment also enhanced both docetaxel and liposomal doxorubicin activity in PC3 tumors [103]. More recent studies have also demonstrated that mice bearing tumors treated with an oncolytic adenovirus expressing PH20 showed more anti tumor efficacy than mice treated with the parental virus alone [104]. These results suggest that PH20 may represent a potentially innovative treatment approach against tumors which produce high levels of HA which includes the majority of ovarian cancers. The company Halozyme has recently commenced a Phase 1 clinical trial which will evaluate a range of doses of PEGPH20 in advanced cancer patients [105].

Thus, HA presents a novel and promising candidate for increasing efficacy and reducing toxicity of cancer therapies. HA conjugates also have a potential role in the development of more effective intra-peritoneal treatment regimes in ovarian cancer [96].

### 4.2. CD44 Therapies

CD44 is also promising target against cancer. Methods which have been used to block the action of CD44 include, neutralizing antibodies, siRNA, antisense RNA, cDNA vaccination as well as the using the naturally occurring CD44-inhibitor silibinin. Silibinin, a natural drug extracted from common thistles, which inhibits CD44 promoter activity and expression, is known to have anti-tumor properties in breast cancer [106], non small cell lung cancer [107], and colorectal carcinoma [108]. It has also been shown to decrease motility and invasion in prostate cancer cells and to decrease adhesion of prostate cancer cells to HA and fibronectin [109].

CD44 cDNA vaccination has been shown to decrease tumor mass and metastatic potential in experimental mammary tumors in mice [110]. In addition, transfection of CD44 antisense RNA into a highly metastatic mammary tumor cell line disrupted expression of CD44 in the tumor cells and reduced their ability to establish local tumors as well as metastatic colonies in the lung [110]. Treatment of gemcitabine resistant pancreatic cancer cells with CD44 siRNA inhibited their proliferative activity [111]. Furthermore, *in vivo* targeting of CD44 by siRNA resulted in anti-tumor activity in both colon cancer and leukemia xenografts [112,113]. In hepatocellular carcinomas, CD44 antisense oligonucleotide significantly down-regulated CD44 expression, induced apoptosis, decreased tumorigenesis and invasion, and increased the cancer cell sensitivity to chemotherapy drugs [114]. In ovarian cancer cells, *in vitro* adhesion, invasion, and resistance to apoptosis were inhibited by CD44 siRNA [115]. Tumor growth and peritoneal dissemination of human ovarian cancer xenografts in nude mice were also decreased.

Another very promising therapeutic approach is the use of anti-CD44 antibodies such as IM7 which resulted in a greater than 50% decrease of HA production in glioma cells and enhanced glioma apoptosis [116]. Treatment of chondrosarcoma cells with IM7 resulted in a significant decrease in cell viability but did not reduce cell viability in normal human chondrocytes [117]. Another antibody against CD44, called P245, inhibited growth of breast cancer xenograft in mice [118]. Affify *et al.* showed that anti-CD44s could inhibit breast cancer cell adhesion, motility and invasion, whilst anti-CD44v6 inhibited cell motility [119]. Furthermore, Guo *et al.* showed that treatment with an anti-CD44 monoclonal antibodies inhibited the formation of melanoma metastases [120] and decreased the invasive ability of breast cancer cells [121]. Treatment of prostate cancer cells with a neutralising CD44 antibody decreased cancer cell adhesion to human bone endothelial cells, a primary site for prostate cancer cell metastasis [122]. In ovarian cancer, Casey *et al.* showed that their CD44 monoclonal antibody did not inhibit ovarian cancer cell invasion but inhibited motility [93], Furthermore, Strobel *et al.* showed a decrease in the number of total peritoneal ovarian cancer metastases in mice when treated with a anti-CD44 antibody [32]. However, despite promising *in vitro* studies, clinical trials with CD44 neutralizing antibodies have been terminated due to unacceptable levels of toxicity [123]. However, with numerous studies finding that disruption of the HA-CD44 interaction decreases the proliferative and metastatic behavior of tumor cells, CD44 still remains a valid target for anti-cancer therapy. Non toxic alternative therapies need to be developed for future trials.

### 4.3. Versican Therapies

Versican levels in the tumor environment can be reduced by inhibition of its production. This has been achieved by treatment with genistein, a tyrosine kinase inhibitor which has been shown to inhibit versican synthesis in vascular smooth muscle cells (SMCs) and malignant mesothelioma cell lines [124,125]. Versican production could also be blocked by the leukotriene receptor antagonist, montelukast, in both bronchial and arterial SMCs [126]. No studies to date have yet investigated whether these inhibitors are effective in inhibiting the effects of versican in cancer models.

More recent studies have demonstrated that drugs used in the treatment of asthma including formoterol, a long acting β_2_-adrenergic agonist, and budesonide, a glucocorticoid steroid, could decrease protein levels of a number of proteoglycans including versican in airway SMCs and human lung fibroblasts [127,128]. Combination treatment with budesonide and formoterol was significantly superior in reducing versican levels than either drug alone [128]. These studies suggest that the changes in versican deposition which occur in cancer tissues, as well as the asthmatic airway and atherosclerotic lesions, could be inhibited by a number of asthma drugs. This, combined with their safe use in humans, makes them potential therapeutic agents against ovarian cancer.

There is increasing evidence that ADAMTS proteases play an important role in the cleavage of versican, and the local accumulation of versican fragments generated by ADAMTS digestion may also promote cancer cell motility and invasion. The broad spectrum MMP inhibitor, GM6001 (Galardin) which inhibits the activity of MMPs and ADAMTS proteases has been shown to inhibit cancer cell invasion and metastasis in several model systems [93,129–131]. One of the more interesting MMP inhibitors is present in green tea, which is made from the leaves of *Camellia sinensis* and contains catechin gallate esters. Catechin gallate esters have been shown to selectively inhibit ADAMTS-1,-4, and -5 and inhibit aggrecan processing in cartilage tissue [132]. The ability of GM6001 and catechin gallate esters to inhibit versican cleavage and versican-induced motility and metastasis of ovarian cancer cells has not been investigated yet. Manipulation of the versican catabolic pathways may provide novel therapeutic targets against cancer invasion and metastasis.

The formation of a pericellular matrix rich in HA and versican by vascular SMCs and tumor cells can be inhibited following treatment with HA oligomers [26,90,133]. Disruption of the HA-CD44 interaction with HA oligomers has been shown to markedly inhibit the growth of melanoma cells [134]. Additionally, both link-TSG6 and G1 domain of aggrecan were shown to bind to polymeric HA and these interactions could be competed with HA-8 and HA-10 oligomers, respectively [135], making it likely that HA oligomers will also block the interaction of versican with HA. Treatment with HA-8 oligomers inhibited the formation of pericellular matrix by osteosarcoma cells and reduced HA accumulation in local tumors, tumor growth, and the formation of distant lung metastases. Furthermore, in osteosarcoma cell lines, both motility and invasiveness were inhibited by the use of HA oligomers [136]. Additionally, colorectal carcinoma growth was reduced both *in vitro* and *in vivo* after treatment with HA oligomers [137]. Our own recent work has shown that HA oligomers can reduce adhesion of ovarian cancer cells to peritoneal cells, and block ovarian cancer motility and invasion induced by versican and HA treatment [26].

HA oligomers have also been shown to reverse chemotherapy resistance in some cancer cell lines [138,139]. Adriamycin resistance of leukemic cells was effectively reversed by HA-4 oligomers by increasing the intracellular accumulation of adriamycin [138], whilst in malignant peripheral nerve sheath tumors (MPNST), HA oligomers resulted in the disassembly of CD44-transporter complexes and induced internalization of CD44 [139]. Furthermore, *in vivo* systemic administration of HA oligomers inhibited the growth of MPNST and ovarian cancer xenografts [139,140]. These findings suggest that the potent anti-tumor effects of HA oligomers are due in part to the blocking of the formation of HA-rich cell-associated matrices. The use of HA oligomers is a potentially attractive reagent to block versican HA interactions as well as local tumor invasion in ovarian cancer but need further *in vivo* investigation.

## 5. Conclusions

Tumor cells interfere with the normal programming of ECM biosynthesis and can extensively modify the structure and composition of the matrix. The peritumoral stroma surrounding ovarian cancer cells is enriched in HA, versican and CD44 all of which can promote ovarian cancer metastatic behavior. These molecules hold promise as therapeutic targets in ovarian cancer and warrant further evaluation.

## Figures and Tables

**Table 1.** Summary of the studies investigating role of HA, hyaluronidase, CD44 and versican effects on epithelial ovarian cancer.

**Molecule**	**Effect**	**References**
**HA**	Ovarian cancer cell bind to HA	[6,18]
	HA is increased at metastatic sites	[19]
	High HA level predicts poor disease outcome	[20]
	HA promotes interaction with CD44v3 and HER2 to induce Rac1 and Ras signalling and increased ovarian cancer migration and proliferation	[21]
	HA and HAS are increased at metastatic sites	[22]
	HA concentration correlates with high tumor grade	[23]
	HA promotes motility of ovarian cancer cells	[14]
	HAS1 levels are associated with reduced survival	[24]
	HA mediates adhesion of ovarian cancer cells to the peritoneum	[25,26]
	HA production by mesothelial cells is increased following co-culture with ovarian cancer cells	[14]

**Hyaluronidase**	Hyaluronidase activity is increased in metastatic gynecological cancers	[27]
	Hyaluronidase HYAL1 is reduced in serous ovarian cancer and results in HA accumulation	[28]
	Hyaluronidase activation is not associated with ovarian cancer aggressiveness	[23]
	HA and hyaluronidase synthesis by ovarian cancer cells is increased by gonadotropins	[25]

**CD44**	CD44 mediates binding peritoneal mesothelium	[13,29,30]
	Patients with CD44v positive tumors have reduced disease-free survival	[31]
	CD44 inhibition limits intra-abdominal spreading	[32]
	CD44-HER2 interactions promote ovarian cancer proliferation	[33]
	High levels of CD44s is an independent predictor of survival	[34]
	Loss of CD44v3 expression is an independent factor for poor survival.	[35]
	CD44 mediates ovarian cancer migration towards ECM	[36]
	CD44 interaction with c-src kinase promotes HA dependent ovarian cancer cell migration	[37]
	CD44s expression relates with tumor stage	[38]
	Decreased CD44s expression correlates with reduced relapse-free survival	[39]
	Increased expression CD44s predicts improved survival	[40]
	High CD44 expression is associated with a favorable prognosis	[41]
	CD44-HA interaction promotes Cdc42 and ERK signaling and ovarian cancer progression	[42]
	CD44-HA interactions promotes cell migration via HER2 activation and beta-catenin nuclear translocation	[43]
	Heregulin mediated ErbB2-ERK signaling activates HAS and CD44 dependent ovarian tumor growth and migration	[44]
	Co-expression of CD44 and multiple drug resistance proteins MDR1 and MDP2 correlates with ovarian cancer progression	[45]

**Versican**	Elevated stromal versican is associated with reduced overall survival	[46]
	Versican is increased in primary and metastatic cancers	[47]
	Versican correlates with metastatic signature	[48]
	Induction of stromal versican expression is associated with tumor progression	[49]
	High stromal versican associates with poorer outcome	[50]
	Versican promotes ovarian cancer migration and invasion	[26,50]

## Abbreviations:

ECM: extracellular matrix
HA: hyaluronan
HAS: hyaluronan synthase
PEI: ployethyenimine
SMCs: smooth muscle cells

## References

[1] Stewart BW, Kleihues P, WHO World Cancer Report (2003).

[2] Jemal A, Siegel R, Ward E, Hao Y, Xu J, Thun MJ (2009). Cancer Statistics, 2009. CA Cancer J Clin.

[3] Amadori D, Sansoni E, Amadori A (1997). Ovarian cancer: natural history and metastatic pattern. Front. Biosci.

[4] Rubin SC, Randall TC, Armstrong KA, Chi DS, Hoskins WJ (1999). Ten-year follow-up of ovarian cancer patients after second-look laparotomy with negative findings. Obstet Gynecol.

[5] Freedman RS, Deavers M, Liu J, Wang E (2004). Peritoneal inflammation—A microenvironment for Epithelial Ovarian Cancer (EOC). J Transl Med.

[6] Gardner MJ, Catterall JB, Jones LM, Turner GA (1996). Human ovarian tumour cells can bind hyaluronic acid via membrane CD44: a possible step in peritoneal metastasis. Clin Exp Metastasis.

[7] Strobel T, Cannistra SA (1999). Beta1-integrins partly mediate binding of ovarian cancer cells to peritoneal mesothelium *in vitro*. Gynecol Oncol.

[8] Said NA, Najwer I, Socha MJ, Fulton DJ, Mok SC, Motamed K (2007). SPARC inhibits LPA-mediated mesothelial-ovarian cancer cell crosstalk. Neoplasia.

[9] Heyman L, Kellouche S, Fernandes J, Dutoit S, Poulain L, Carreiras F (2008). Vitronectin and its receptors partly mediate adhesion of ovarian cancer cells to peritoneal mesothelium *in vitro*. Tumour Biol.

[10] Kenny HA, Kaur S, Coussens LM, Lengyel E (2008). The initial steps of ovarian cancer cell metastasis are mediated by MMP-2 cleavage of vitronectin and fibronectin. J Clin Invest.

[11] Ween MP, Lokman NA, Hoffmann P, Rodgers RJ, Ricciardelli C, Oehler MK (2010). Transforming growth factor beta-induced protein (TGFBIp) secreted by peritoneal cells increases the metastatic potential of ovarian cancer cells. Int J Cancer.

[12] Jones LM, Gardner MJ, Catterall JB, Turner GA (1995). Hyaluronic acid secreted by mesothelial cells: a natural barrier to ovarian cancer cell adhesion. Clin Exp Metastasis.

[13] Lessan K, Aguiar DJ, Oegema T, Siebenson L, Skubitz AP (1999). CD44 and beta1 integrin mediate ovarian carcinoma cell adhesion to peritoneal mesothelial cells. Am J Pathol.

[14] Carpenter PM, Dao AV (2003). The role of hyaluronan in mesothelium-induced motility of ovarian carcinoma cells. Anticancer Res.

[15] Zigrino P, Loffek S, Mauch C (2005). Tumor-stroma interactions: their role in the control of tumor cell invasion. Biochimie.

[16] Liotta LA, Kohn EC (2001). The microenvironment of the tumour-host interface. Nature.

[17] Ricciardelli C, Rodgers RJ (2006). Extracellular matrix of ovarian tumors. Semin Reprod Med.

[18] Catterall JB, Gardner MJ, Jones LM, Turner GA (1997). Binding of ovarian cancer cells to immobilized hyaluronic acid. Glycoconj J.

[19] Yeo TK, Nagy JA, Yeo KT, Dvorak HF, Toole BP (1996). Increased hyaluronan at sites of attachment to mesentery by CD44-positive mouse ovarian and breast tumor cells. Am J Pathol.

[20] Anttila MA, Tammi RH, Tammi MI, Syrjanen KJ, Saarikoski SV, Kosma VM (2000). High levels of stromal hyaluronan predict poor disease outcome in epithelial ovarian cancer. Cancer Res.

[21] Bourguignon LY, Zhu H, Zhou B, Diedrich F, Singleton PA, Hung MC (2001). Hyaluronan promotes CD44v3-Vav2 interaction with Grb2-p185(HER2) and induces Rac1 and Ras signaling during ovarian tumor cell migration and growth. J Biol Chem.

[22] Jojovic M, Delpech B, Prehm P, Schumacher U (2002). Expression of hyaluronate and hyaluronate synthase in human primary tumours and their metastases in scid mice. Cancer Lett.

[23] Hiltunen EL, Anttila M, Kultti A, Ropponen K, Penttinen J, Yliskoski M, Kuronen AT, Juhola M, Tammi R, Tammi M, Kosma VM (2002). Elevated hyaluronan concentration without hyaluronidase activation in malignant epithelial ovarian tumors. Cancer Res.

[24] Yabushita H, Noguchi M, Kishida T, Fusano K, Noguchi Y, Itano N, Kimata K (2004). Hyaluronan synthase expression in ovarian cancer. Oncol Rep.

[25] Tzuman YC, Sapoznik S, Granot D, Nevo N, Neeman M (2010). Peritoneal adhesion and angiogenesis in ovarian carcinoma are inversely regulated by hyaluronan: the role of gonadotropins. Neoplasia.

[26] Ween MP, Hummitzsch K, Rodgers RJ, Oehler MK, Ricciardelli C (2010). Versican induces a pro-metastatic ovarian cancer cell behavior which can be inhibited by small hyaluronan oligosaccharides. Clin Exp Metastasis.

[27] Tamakoshi K, Kikkawa F, Maeda O, Suganuma N, Yamagata S, Yamagata T, Tomoda Y (1997). Hyaluronidase activity in gynaecological cancer tissues with different metastatic forms. Br J Cancer.

[28] Nykopp TK, Rilla K, Sironen R, Tammi MI, Tammi RH, Hamalainen K, Heikkinen AM, Komulainen M, Kosma VM, Anttila M (2009). Expression of hyaluronan synthases (HAS1-3) and hyaluronidases (HYAL1-2) in serous ovarian carcinomas: inverse correlation between HYAL1 and hyaluronan content. BMC Cancer.

[29] Cannistra SA, Kansas GS, Niloff J, DeFranzo B, Kim Y, Ottensmeier C (1993). Binding of ovarian cancer cells to peritoneal mesothelium *in vitro* is partly mediated by CD44H. Cancer Res.

[30] Gardner MJ, Jones LM, Catterall JB, Turner GA (1995). Expression of cell adhesion molecules on ovarian tumour cell lines and mesothelial cells, in relation to ovarian cancer metastasis. Cancer Lett.

[31] Uhl-Steidl M, Muller-Holzner E, Zeimet AG, Adolf GR, Daxenbichler G, Marth C, Dapunt O (1995). Prognostic value of CD44 splice variant expression in ovarian cancer. Oncology.

[32] Strobel T, Swanson L, Cannistra SA (1997). *In vivo* inhibition of CD44 limits intra-abdominal spread of a human ovarian cancer xenograft in nude mice: a novel role for CD44 in the process of peritoneal implantation. Cancer Res.

[33] Bourguignon LY, Zhu H, Chu A, Iida N, Zhang L, Hung MC (1997). Interaction between the adhesion receptor, CD44, and the oncogene product, p185HER2, promotes human ovarian tumor cell activation. J Biol Chem.

[34] Kayastha S, Freedman AN, Piver MS, Mukkamalla J, Romero-Guittierez M, Werness BA (1999). Expression of the hyaluronan receptor, CD44S, in epithelial ovarian cancer is an independent predictor of survival. Clin Cancer Res.

[35] Saegusa M, Machida D, Hashimura M, Okayasu I (1999). CD44 expression in benign, premalignant, and malignant ovarian neoplasms: relation to tumour development and progression. J Pathol.

[36] Casey RC, Skubitz AP (2000). CD44 and beta1 integrins mediate ovarian carcinoma cell migration toward extracellular matrix proteins. Clin Exp Metastasis.

[37] Bourguignon LY, Zhu H, Shao L, Chen YW (2001). CD44 interaction with c-Src kinase promotes cortactin-mediated cytoskeleton function and hyaluronic acid-dependent ovarian tumor cell migration. J Biol Chem.

[38] Afify AM, Ferguson AW, Davila RM, Werness BA (2001). Expression of CD44S and CD44v5 is more common in stage III than in stage I serous ovarian carcinomas. Appl Immunohistochem Mol Morphol.

[39] Ross JS, Sheehan CE, Williams SS, Malfetano JH, Szyfelbein WM, Kallakury BV (2001). Decreased CD44 standard form expression correlates with prognostic variables in ovarian carcinomas. Am J Clin Pathol.

[40] Rodriguez-Rodriguez L, Sancho-Torres I, Mesonero C, Gibbon DG, Shih WJ, Zotalis G (2003). The CD44 receptor is a molecular predictor of survival in ovarian cancer. Med Oncol.

[41] Sillanpaa S, Anttila MA, Voutilainen K, Tammi RH, Tammi MI, Saarikoski SV, Kosma VM (2003). CD44 expression indicates favorable prognosis in epithelial ovarian cancer. Clin Cancer Res.

[42] Bourguignon LY, Gilad E, Rothman K, Peyrollier K (2005). Hyaluronan-CD44 interaction with IQGAP1 promotes Cdc42 and ERK signaling, leading to actin binding, Elk-1/estrogen receptor transcriptional activation, and ovarian cancer progression. J Biol Chem.

[43] Bourguignon LY, Peyrollier K, Gilad E, Brightman A (2007). Hyaluronan-CD44 interaction with neural Wiskott-Aldrich syndrome protein (N-WASP) promotes actin polymerization and ErbB2 activation leading to beta-catenin nuclear translocation, transcriptional up-regulation, and cell migration in ovarian tumor cells. J Biol Chem.

[44] Bourguignon LY, Gilad E, Peyrollier K (2007). Heregulin-mediated ErbB2-ERK signaling activates hyaluronan synthases leading to CD44-dependent ovarian tumor cell growth and migration. J Biol Chem.

[45] Chen H, Hao J, Wang L, Li Y (2009). Coexpression of invasive markers (uPA, CD44) and multiple drug-resistance proteins (MDR1, MRP2) is correlated with epithelial ovarian cancer progression. Br J Cancer.

[46] Voutilainen K, Anttila M, Sillanpaa S, Tammi R, Tammi M, Saarikoski S, Kosma VM (2003). Versican in epithelial ovarian cancer: Relation to hyaluronan, clinicopathologic factors and prognosis. Int J Cancer.

[47] Casey RC, Oegema TR (2003). ; Skubitz, K.M.; Pambuccian, S.E.; Grindle, S.M.; Skubitz, A.P. Cell membrane glycosylation mediates the adhesion, migration, and invasion of ovarian carcinoma cells. Clin Exp Metastasis.

[48] Lancaster JM, Dressman HK, Clarke JP, Sayer RA, Martino MA, Cragun JM, Henriott AH, Gray J, Sutphen R, Elahi A, Whitaker RS, West M, Marks JR, Nevins JR, Berchuck A (2006). Identification of genes associated with ovarian cancer metastasis using microarray expression analysis. Int J Gynecol Cancer.

[49] Kusumoto T, Kodama J, Seki N, Nakamura K, Hongo A, Hiramatsu Y (2010). Clinical significance of syndecan-1 and versican expression in human epithelial ovarian cancer. Oncol Rep.

[50] Ghosh S, Albitar L, Lebaron R, Welch WR, Samimi G, Birrer MJ, Berkowitz RS, Mok SC (2010). Up-regulation of stromal versican expression in advanced stage serous ovarian cancer. Gynecol. Oncol.

[51] Knudson CB, Knudson W (1993). Hyaluronan-binding proteins in development, tissue homeostasis, and disease. FASEB J.

[52] Weigel PH, Hascall VC, Tammi M (1997). Hyaluronan synthases. J Biol Chem.

[53] Tammi MI, Day AJ, Turley EA (2002). Hyaluronan and homeostasis: a balancing act. J Biol Chem.

[54] Toole BP (2004). Hyaluronan: from extracellular glue to pericellular cue. Nat Rev Cancer.

[55] Boregowda RK, Appaiah HN, Siddaiah M, Kumarswamy SB, Sunila S, Thimmaiah KN, Mortha K, Toole B, Banerjee S (2006). Expression of hyaluronan in human tumor progression. J. Carcinog.

[56] Auvinen P, Tammi R, Parkkinen J, Tammi M, Agren U, Johansson R, Hirvikoski P, Eskelinen M, Kosma VM (2000). Hyaluronan in peritumoral stroma and malignant cells associates with breast cancer spreading and predicts survival. Am J Pathol.

[57] Ropponen K, Tammi M, Parkkinen J, Eskelinen M, Tammi R, Lipponen P, Agren U, Alhava E, Kosma VM (1998). Tumor cell-associated hyaluronan as an unfavorable prognostic factor in colorectal cancer. Cancer Res.

[58] Lipponen P, Aaltomaa S, Tammi R, Tammi M, Agren U, Kosma VM (2001). High stromal hyaluronan level is associated with poor differentiation and metastasis in prostate cancer. Eur J Cancer.

[59] Pirinen R, Tammi R, Tammi M, Hirvikoski P, Parkkinen JJ, Johansson R, Bohm J, Hollmen S, Kosma VM (2001). Prognostic value of hyaluronan expression in non-small-cell lung cancer: Increased stromal expression indicates unfavorable outcome in patients with adenocarcinoma. Int J Cancer.

[60] Posey JT, Soloway MS, Ekici S, Sofer M, Civantos F, Duncan RC, Lokeshwar VB (2003). Evaluation of the prognostic potential of hyaluronic acid and hyaluronidase (HYAL1) for prostate cancer. Cancer Res.

[61] Aruffo A, Stamenkovic I, Melnick M, Underhill CB, Seed B (1990). CD44 is the principal cell surface receptor for hyaluronate. Cell.

[62] Bourguignon LY, Zhu D, Zhu H (1998). CD44 isoform-cytoskeleton interaction in oncogenic signaling and tumor progression. Front. Biosci.

[63] Screaton GR, Bell MV, Jackson DG, Cornelis FB, Gerth U, Bell JI (1992). Genomic structure of DNA encoding the lymphocyte homing receptor CD44 reveals at least 12 alternatively spliced exons. Proc Natl Acad Sci USA.

[64] Naor D, Nedvetzki S, Golan I, Melnik L, Faitelson Y (2002). CD44 in cancer. Crit Rev Clin Lab Sci.

[65] Stickeler E, Runnebaum IB, Mobus VJ, Kieback DG, Kreienberg R (1997). Expression of CD44 standard and variant isoforms v5, v6 and v7 in human ovarian cancer cell lines. Anticancer Res.

[66] Zhu D, Bourguignon LY (2000). Interaction between CD44 and the repeat domain of ankyrin promotes hyaluronic acid-mediated ovarian tumor cell migration. J Cell Physiol.

[67] Bourguignon LY, Zhu H, Shao L, Chen YW (2000). Ankyrin-Tiam1 interaction promotes Rac1 signaling and metastatic breast tumor cell invasion and migration. J Cell Biol.

[68] Kim Y, Lee YS, Choe J, Lee H, Kim YM, Jeoung D (2008). CD44-epidermal growth factor receptor interaction mediates hyaluronic acid-promoted cell motility by activating protein kinase C signaling involving Akt, Rac1, Phox, reactive oxygen species, focal adhesion kinase, and MMP-2. J Biol Chem.

[69] Buess M, Rajski M, Vogel-Durrer BM, Herrmann R, Rochlitz C (2009). Tumor-endothelial interaction links the CD44(+)/CD24(−) phenotype with poor prognosis in early-stage breast cancer. Neoplasia.

[70] Okayama H, Kumamoto K, Saitou K, Hayase S, Kofunato Y, Sato Y, Miyamoto K, Nakamura I, Ohki S, Sekikawa K, Takenoshita S (2009). CD44v6, MMP-7 and nuclear Cdx2 are significant biomarkers for prediction of lymph node metastasis in primary gastric cancer. Oncol Rep.

[71] Kawano T, Nakamura Y, Yanoma S, Kubota A, Furukawa M, Miyagi Y, Tsukuda M (2004). Expression of E-cadherin, and CD44s and CD44v6 and its association with prognosis in head and neck cancer. Auris Nasus Larynx.

[72] Lee SM, Lee KE, Chang HJ, Choi MY, Cho MS, Min SK, Lee HK, Mun YC, Nam EM, Seong CM, Lee SN (2008). Prognostic significance of CD44s expression in biliary tract cancers. Ann Surg Oncol.

[73] Gu H, Shang P, Zhou C (2004). Expression of CD44v6 and E-cadherin in prostate carcinoma and metastasis of prostate carcinoma. Zhonghua Nan Ke Xue.

[74] Cannistra SA, Abu-Jawdeh G, Niloff J, Strobel T, Swanson L, Andersen J, Ottensmeier C (1995). CD44 variant expression is a common feature of epithelial ovarian cancer: lack of association with standard prognostic factors. J Clin Oncol.

[75] Zagorianakou N, Stefanou D, Makrydimas G, Zagorianakou P, Briasoulis E, Karavasilis B, Agnantis NJ (2004). CD44s expression, in benign, borderline and malignant tumors of ovarian surface epithelium. Correlation with p53, steroid receptor status, proliferative indices (PCNA, MIB1) and survival. Anticancer Res.

[76] Du Cros DL, Lebaron RG, Couchman JR (1995). Association of versican with dermal matrices and its potential role in hair follicle development and cycling. J Invest Dermatol.

[77] Suwiwat S, Ricciardelli C, Tammi R, Tammi M, Auvinen P, Kosma VM, LeBaron RG, Raymond WA, Tilley WD, Horsfall DJ (2004). Expression of extracellular matrix components versican, chondroitin sulfate, tenascin, and hyaluronan, and their association with disease outcome in node-negative breast cancer. Clin Cancer Res.

[78] Speiser P, Wanner C, Breitenecker G, Kohlberger P, Kainz C (1995). CD-44 is not involved in the metastatic spread of ovarian cancer *in vivo*. Anticancer Res.

[79] Sanchez Lockhart M, Hajos SE, Basilio FM, Mongini C, Alvarez E (2001). Splice variant expression of CD44 in patients with breast and ovarian cancer. Oncol Rep.

[80] Ricciardelli C, Sakko AJ, Ween MP, Russell DL, Horsfall DJ (2009). The biological role and regulation of versican levels in cancer. Cancer Metastasis Rev.

[81] LeBaron RG (1996). Versican. Perspect Dev Neurobiol.

[82] Zimmermann DR, Ruoslahti E (1989). Multiple domains of the large fibroblast proteoglycan, versican. EMBO J.

[83] Wu YJ, La Pierre DP, Wu J, Yee AJ, Yang BB (2005). The interaction of versican with its binding partners. Cell Res.

[84] Naso MF, Zimmermann DR, Iozzo RV (1994). Characterization of the complete genomic structure of the human versican gene and functional analysis of its promoter. J BiolChem.

[85] Kischel P, Waltregny D, Dumont B, Turtoi A, Greffe Y, Kirsch S, de Pauw E, Castronovo V (2010). Versican overexpression in human breast cancer lesions: known and new isoforms for stromal tumor targeting. Int J Cancer.

[86] Sakko AJ, Ricciardelli C, Mayne K, Tilley WD, LeBaron RG, Horsfall DJ (2001). Versican accumulation in human prostatic fibroblast cultures is enhanced by prostate cancer cell-derived transforming growth factor beta1. Cancer Res.

[87] Ricciardelli C, Brooks JH, Suwiwat S, Sakko AJ, Mayne K, Raymond WA, Seshadri R, LeBaron RG, Horsfall DJ (2002). Regulation of stromal versican expression by breast cancer cells and importance to relapse-free survival in patients with node-negative primary breast cancer. Clin Cancer Res.

[88] Ghosh S, Albitar L, LeBaron R, Welch WR, Samimi G, Birrer MJ, Berkowitz RS, Mok SC (2010). Up-regulation of stromal versican expression in advanced stage serous ovarian cancer. Gynecol Oncol.

[89] Knudson W, Bartnik E, Knudson CB (1993). Assembly of pericellular matrices by COS-7 cells transfected with CD44 lymphocyte-homing receptor genes. Proc Natl Acad Sci USA.

[90] Evanko SP, Angello JC, Wight TN (1999). Formation of hyaluronan- and versican-rich pericellular matrix is required for proliferation and migration of vascular smooth muscle cells. Arterioscler Thromb Vasc Biol.

[91] Ricciardelli C, Russell DL, Ween MP, Mayne K, Suwiwat S, Byers S, Marshall VR, Tilley WD, Horsfall DJ (2007). Formation of hyaluronan- and versican-rich pericellular matrix by prostate cancer cells promotes cell motility. J Biol Chem.

[92] Casey MJ, Bewtra C, Hoehne LL, Tatpati AD, Lynch HT, Watson P (2000). Histology of prophylactically removed ovaries from BRCA1 and BRCA2 mutation carriers compared with noncarriers in hereditary breast ovarian cancer syndrome kindreds. Gynecol Oncol.

[93] Casey RC, Koch KA, Oegema TR, Skubitz KM, Pambuccian SE, Grindle SM, Skubitz AP (2003). Establishment of an *in vitro* assay to measure the invasion of ovarian carcinoma cells through mesothelial cell monolayers. Clin Exp Metastasis.

[94] Lee H, Lee K, Park TG (2008). Hyaluronic acid-paclitaxel conjugate micelles: synthesis, characterization, and antitumor activity. Bioconjug Chem.

[95] Cohen MS, Cai S, Xie Y, Forrest ML (2009). A novel intralymphatic nanocarrier delivery system for cisplatin therapy in breast cancer with improved tumor efficacy and lower systemic toxicity *in vivo*. Am J Surg.

[96] Banzato A, Bobisse S, Rondina M, Renier D, Bettella F, Esposito G, Quintieri L, Melendez-Alafort L, Mazzi U, Zanovello P, Rosato A (2008). A paclitaxel-hyaluronan bioconjugate targeting ovarian cancer affords a potent *in vivo* therapeutic activity. Clin Cancer Res.

[97] Auzenne E, Ghosh SC, Khodadadian M, Rivera B, Farquhar D, Price RE, Ravoori M, Kundra V, Freedman RS, Klostergaard J (2007). Hyaluronic acid-paclitaxel: antitumor efficacy against CD44(+) human ovarian carcinoma xenografts. Neoplasia.

[98] Sun X, Ma P, Cao X, Ning L, Tian Y, Ren C (2009). Positive hyaluronan/PEI/DNA complexes as a target-specific intracellular delivery to malignant breast cancer. Drug Deliv.

[99] Jiang G, Park K, Kim J, Kim KS, Hahn SK (2009). Target specific intracellular delivery of siRNA/PEI-HA complex by receptor mediated endocytosis. Mol Pharm.

[100] Chono S, Li SD, Conwell CC, Huang L (2008). An efficient and low immunostimulatory nanoparticle formulation for systemic siRNA delivery to the tumor. J Control Release.

[101] Baumgartner G, Gomar-Hoss C, Sakr L, Ulsperger E, Wogritsch C (1998). The impact of extracellular matrix on the chemoresistance of solid tumors—experimental and clinical results of hyaluronidase as additive to cytostatic chemotherapy. Cancer Lett.

[102] Maier U, Baumgartner G (1989). Metaphylactic effect of mitomycin C with and without hyaluronidase after transurethral resection of bladder cancer: randomized trial. J Urol.

[103] Thompson CB, Shepard HM, O’Connor PM, Kadhim S, Jiang P, Osgood RJ, Bookbinder LH, Li X, Sugarman BJ, Connor RJ, Nadjsombati S, Frost GI (2010). Enzymatic depletion of tumor hyaluronan induces antitumor responses in preclinical animal models. Mol Cancer Ther.

[104] Guedan S, Rojas JJ, Gros A, Mercade E, Cascallo M, Alemany R (2010). Hyaluronidase expression by an oncolytic adenovirus enhances its intratumoral spread and suppresses tumor growth. Mol Ther.

[105] Homepage of Halozyme http://www.halozyme.com/products_oncology.php.

[106] Lee SO, Jeong YJ, Im HG, Kim CH, Chang YC, Lee IS (2007). Silibinin suppresses PMAinduced MMP-9 expression by blocking the AP-1 activation via MAPK signaling pathways in MCF-7 human breast carcinoma cells. Biochem Biophys Res Commun.

[107] Mateen S, Tyagi A, Agarwal C, Singh RP, Agarwal R (2010). Silibinin inhibits human nonsmall cell lung cancer cell growth through cell-cycle arrest by modulating expression and function of key cell-cycle regulators. Mol Carcinog.

[108] Kaur M, Velmurugan B, Tyagi A, Deep G, Katiyar S, Agarwal C, Agarwal R (2009). Silibinin suppresses growth and induces apoptotic death of human colorectal carcinoma LoVo cells in culture and tumor xenograft. Mol Cancer Ther.

[109] Handorean AM, Yang K, Robbins EW, Flaig TW, Iczkowski KA (2009). Silibinin suppresses CD44 expression in prostate cancer cells. Am J Transl Res.

[110] Wallach-Dayan SB, Rubinstein AM, Hand C, Breuer R, Naor D (2008). DNA vaccination with CD44 variant isoform reduces mammary tumor local growth and lung metastasis. Mol Cancer Ther.

[111] Hong SP, Wen J, Bang S, Park S, Song SY (2009). CD44-positive cells are responsible for gemcitabine resistance in pancreatic cancer cells. Int J Cancer.

[112] Jin L, Hope KJ, Zhai Q, Smadja-Joffe F, Dick JE (2006). Targeting of CD44 eradicates human acute myeloid leukemic stem cells. Nat Med.

[113] Subramaniam V, Vincent IR, Gilakjan M, Jothy S (2007). Suppression of human colon cancer tumors in nude mice by siRNA CD44 gene therapy. Exp Mol Pathol.

[114] Xie Z, Choong PF, Poon LF, Zhou J, Khng J, Jasinghe VJ, Palaniyandi S, Chen CS (2008). Inhibition of CD44 expression in hepatocellular carcinoma cells enhances apoptosis, chemosensitivity, and reduces tumorigenesis and invasion. Cancer Chemother Pharmacol.

[115] Li CZ, Liu B, Wen ZQ, Li HY (2008). Inhibition of CD44 expression by small interfering RNA to suppress the growth and metastasis of ovarian cancer cells *in vitro* and *in vivo*. Folia Biol. (Praha).

[116] Wiranowska M, Ladd S, Moscinski LC, Hill B, Haller E, Mikecz K, Plaas A (2010). Modulation of Hyaluronan production by CD44 positive Glioma cells. Int J Cancer.

[117] Yoshida M, Yasuda T, Hiramitsu T, Ito H, Nakamura T (2008). Induction of apoptosis by anti-CD44 antibody in human chondrosarcoma cell line SW1353. Biomed Res.

[118] Marangoni E, Lecomte N, Durand L, de Pinieux G, Decaudin D, Chomienne C, Smadja-Joffe F, Poupon MF (2009). CD44 targeting reduces tumour growth and prevents post-chemotherapy relapse of human breast cancers xenografts. Br J Cancer.

[119] Afify A, Purnell P, Nguyen L (2009). Role of CD44s and CD44v6 on human breast cancer cell adhesion, migration, and invasion. Exp Mol Pathol.

[120] Guo Y, Ma J, Wang J, Che X, Narula J, Bigby M, Wu M, Sy MS (1994). Inhibition of human melanoma growth and metastasis *in vivo* by anti-CD44 monoclonal antibody. Cancer Res.

[121] Herrera-Gayol A, Jothy S (1999). CD44 modulates Hs578T human breast cancer cell adhesion, migration, and invasiveness. Exp Mol Pathol.

[122] Draffin JE, McFarlane S, Hill A, Johnston PG, Waugh DJ (2004). CD44 potentiates the adherence of metastatic prostate and breast cancer cells to bone marrow endothelial cells. Cancer Res.

[123] Orian-Rousseau V (2010). CD44, a therapeutic target for metastasising tumours. Eur J Cancer.

[124] Schonherr E, Kinsella MG, Wight TN (1997). Genistein selectively inhibits platelet-derived growth factor-stimulated versican biosynthesis in monkey arterial smooth muscle cells. Arch Biochem Biophys.

[125] Syrokou A, Tzanakakis GN, Hjerpe A, Karamanos NK (1999). Proteoglycans in human malignant mesothelioma. Stimulation of their synthesis induced by epidermal, insulin and platelet-derived growth factors involves receptors with tyrosine kinase activity. Biochimie.

[126] Potter-Perigo S, Baker C, Tsoi C, Braun KR, Isenhath S, Altman GM, Altman LC, Wight TN (2004). Regulation of proteoglycan synthesis by leukotriene d4 and epidermal growth factor in bronchial smooth muscle cells. Am J Respir Cell Mol Biol.

[127] Burgess JK, Oliver BG, Poniris MH, Ge Q, Boustany S, Cox N, Moir LM, Johnson PR, Black JL (2006). A phosphodiesterase 4 inhibitor inhibits matrix protein deposition in airways *in vitro*. J Allergy Clin Immunol.

[128] Todorova L, Gurcan E, Miller-Larsson A, Westergren-Thorsson G (2006). Lung fibroblast proteoglycan production induced by serum is inhibited by budesonide and formoterol. Am J Respir Cell Mol Biol.

[129] Arslan F, Bosserhoff AK, Nickl-Jockschat T, Doerfelt A, Bogdahn U, Hau P (2007). The role of versican isoforms V0/V1 in glioma migration mediated by transforming growth factor-beta2. Br J Cancer.

[130] Nakamura JL, Haas-Kogan DA, Pieper RO (2007). Glioma invasiveness responds variably to irradiation in a co-culture model. Int J Radiat Oncol Biol Phys.

[131] Almholt K, Juncker-Jensen A, Laerum OD, Dano K, Johnsen M, Lund LR, Romer J (2008). Metastasis is strongly reduced by the matrix metalloproteinase inhibitor Galardin in the MMTV-PymT transgenic breast cancer model. Mol Cancer Ther.

[132] Vankemmelbeke MN, Jones GC, Fowles C, Ilic MZ, Handley CJ, Day AJ, Knight CG, Mort JS, Buttle DJ (2003). Selective inhibition of ADAMTS-1, -4 and -5 by catechin gallate esters. Eur J Biochem.

[133] Knudson W, Knudson CB (1991). Assembly of a chondrocyte-like pericellular matrix on non-chondrogenic cells. Role of the cell surface hyaluronan receptors in the assembly of a pericellular matrix. J Cell Sci.

[134] Zeng C, Toole BP, Kinney SD, Kuo JW, Stamenkovic I (1998). Inhibition of tumor growth *in vivo* by hyaluronan oligomers. Int J Cancer.

[135] Heng BC, Gribbon PM, Day AJ, Hardingham TE (2008). Hyaluronan binding to link module of TSG-6 and to G1 domain of aggrecan is differently regulated by pH. J Biol Chem.

[136] Hosono K, Nishida Y, Knudson W, Knudson CB, Naruse T, Suzuki Y, Ishiguro N (2007). Hyaluronan oligosaccharides inhibit tumorigenicity of osteosarcoma cell lines MG-63 and LM-8 *in vitro* and *in vivo* via perturbation of hyaluronan-rich pericellular matrix of the cells. Am J Pathol.

[137] Alaniz L, Rizzo M, Malvicini M, Jaunarena J, Avella D, Atorrasagasti C, Aquino JB, Garcia M, Matar P, Silva M, Mazzolini G (2009). Low molecular weight hyaluronan inhibits colorectal carcinoma growth by decreasing tumor cell proliferation and stimulating immune response. Cancer Lett.

[138] Cui X, Zhou S, Xu H, Zhao T, Liu A, Guo X, Wang F (2009). Reversal effects of hyaluronan oligosaccharides on adriamycin resistance of K562/A02 cells. Anticancer Drugs.

[139] Slomiany MG, Dai L, Bomar PA, Knackstedt TJ, Kranc DA, Tolliver L, Maria BL, Toole BP (2009). Abrogating drug resistance in malignant peripheral nerve sheath tumors by disrupting hyaluronan-CD44 interactions with small hyaluronan oligosaccharides. Cancer Res.

[140] Slomiany MG, Dai L, Tolliver LB, Grass GD, Zeng Y, Toole BP (2009). Inhibition of Functional Hyaluronan-CD44 Interactions in CD133-positive Primary Human Ovarian Carcinoma Cells by Small Hyaluronan Oligosaccharides. Clin Cancer Res.

